# Gender-Specific Differences in Spinal Alignment and Muscle Power in Patients with Parkinson’s Disease

**DOI:** 10.3390/diagnostics14111143

**Published:** 2024-05-30

**Authors:** Luciano Bissolotti, Matteo Rota, Stefano Calza, Carlos Romero-Morales, José Luís Alonso-Pérez, Rubén López-Bueno, Jorge Hugo Villafañe

**Affiliations:** 1 Fondazione Teresa Camplani Casa di Cura Domus Salutis, 25123 Brescia, Italy; luciano.bissolotti@fondazioncamplani.it; 2 Department of Molecular and Translational Medicine, University of Brescia, 25121 Brescia, Italy; matteo.rota@unibs.it (M.R.); stefano.calza@unibs.it (S.C.); 3 Department of Physiotherapy, Faculty of Sport Sciences, Universidad Europea de Madrid, 28670 Villaviciosa de Odón, Spain; carlos.romero@universidadeuropea.es; 4 Musculoskeletal Pain and Motor Control Research Group, Faculty of Sport Sciences, Universidad Europea de Madrid, 28670 Villaviciosa de Odón, Spain; 5 Musculoskeletal Pain and Motor Control Research Group, Faculty of Health Sciences, Universidad Europea de Canarias, C/Inocencio García 1, 38300 La Orotava, Canary Islands, Spain; 6 Department of Physiotherapy, Faculty of Health Sciences, Universidad Europea de Canarias, 38300 Santa Cruz de Tenerife, Spain; 7 Onelife Center, Multidisciplinary Pain Treatment Center, 28925 Alcorcón, Spain; 8 Department of Physical Medicine and Nursing, University of Zaragoza, 50009 Zaragoza, Spain; rlopezbu@unizar.es; 9 National Research Centre for the Working Environment, 2100 Copenhagen, Denmark; 10 Exercise Intervention for Health Research Group (EXINH-RG), Department of Physiotherapy, University of Valencia, 46100 Valencia, Spain

**Keywords:** Parkinson’s disease, lower limb, spine

## Abstract

Background: Parkinson’s disease (PD) is an advancing neurodegenerative disorder characterized by spinal anomalies and muscular weakness, which may restrict daily functional capacities. A gender-focused examination of these effects could provide valuable insights into customized rehabilitation strategies for both sexes. Purpose: This study investigates the influence of spinal alignment on lower-limb function during the sit-to-stand (STS) movement in patients with Parkinson’s disease compared to healthy individuals. Methods: A cross-sectional study was conducted with 43 consecutive patients with PD (25 males and 18 females; average age 73.7 ± 7.1 years) and 42 healthy controls (22 males and 20 females; average age 69.8 ± 6.0 years). Assessments included the International Physical Activity Questionnaire (IPAQ), Hoehn and Yahr staging, and measurements of vertical deviations from several spinal landmarks. Lower-limb muscle power during the STS task was evaluated using the Muscle Quality Index (MQI). Results: Both absolute (Watts) and relative (Watts/Kg) muscle power in the lower limbs were notably decreased in the PD group compared to the control group. Within the PD cohort, muscle power showed a negative relationship with age and a positive association with the degree of lumbar lordosis (PL-L3). Importantly, gender-specific analysis revealed that male patients with PD had significantly higher lower-limb muscle power compared to female patients with PD, highlighting the need for gender-tailored therapeutic approaches. Conclusions: The findings suggest that preserving lumbar lordosis is crucial for maintaining effective lower-limb muscle biomechanics in individuals with Parkinson’s disease.

## 1. Introduction

Individuals affected by Parkinson’s disease (PD) often exhibit abnormal postures; previous studies have observed deformities in the limbs, neck, or trunk among these patients [1,2]. One of the most frequently observed deformities is the characteristic stooped posture, involving flexion of the hips and knees with rounded shoulders. However, a significant group of these patients presents more severe postural abnormalities on the sagittal or frontal planes, including camptocormia, antecollis, Pisa syndrome, and scoliosis [3,4]. These severe postural deformities are usually linked to considerable disabilities and have a multifactorial etiology [5].

Contributing factors to these deformities include muscular rigidity, axial dystonia, muscle weakness due to myopathy, body schema defects due to centrally impaired proprioception, and structural changes in the spine [6,7]. The relative importance of these factors varies among patients, complicating their management. Moreover, it is widely recognized that functional deterioration accompanies deterioration of the joints and spine [4,8].

It has been noted in literature that individuals with Parkinson’s disease (PD) often experience weakened lower-limb muscles, impacting their strength and power, which, in turn, can affect their ability to perform daily activities independently [9,10]. Assessing performance in tasks such as repeated sit-to-stand (STS) exercises holds clinical relevance due to its association with fall risk and its cost-effective nature in evaluating lower-limb strength and power [11].

While previous studies have investigated aspects of posture, function, and fall risk in patients with PD, there appears to be a gap in the literature regarding the relationship between spinal alignment and lower-limb performance during repeated STS movements. To address this gap, we employed the Muscle Quality Index (MQI) test [12], specifically chosen for its ability to evaluate lower-limb function, which is particularly relevant in patients with PD for whom such functions are often compromised [12]. The MQI test involves a time-based assessment wherein patients are asked to rise from a chair without using their hands, with the recorded time in seconds serving as the test outcome.

Assessment of spine deformities in patients with PD typically involves clinical or radiographic parameters, some of which correlate significantly with sagittal balance as observed on X-ray examination [3,13,14,15].

The clinical assessment of morphologic spinal maladaptive changes can be assessed during clinical examinations adopting linear and angular measures. The linear measures on the sagittal plane are represented by plumblines distances from the spinous processes of seventh cervical vertebrae (PL-C7), kyphosis apex (PL-AK), and lumbar concavity at the third lumbar vertebrae (PL-L3), as previously published in other papers [16,17,18,19]. On the same plane, the angular measures can be derived using digital inclinometer [18,20].

The critical analysis of spinal alignment in patients with PD and its relationship with lower-limb functionality are not solely academic concerns but have practical implications for clinical practices and patient outcomes. Studies have demonstrated that postural impairments in PD are linked with increased morbidity and reduced quality of life, necessitating comprehensive management strategies that address both the neurological and musculoskeletal aspects of the disease.

In this study, we aim to deepen the understanding of these relationships by examining the association between spinal alignment and the functional capacity of the lower limbs during dynamic tasks such as the STS. By doing so, we hope to identify potential therapeutic targets that could mitigate the musculoskeletal impacts of PD, enhance patient autonomy, and improve overall quality of life. The specificity of sex-related differences in spinal alignment and muscle power during these tasks will also be explored, adding a dimension of personalized medicine to our approach.

Overall, this paper intends to bridge the gap between observational data and clinical application, providing insights that could lead to improved diagnostic accuracy and more effective, tailored interventions for individuals suffering from Parkinson’s disease.

## 2. Materials and Methods

### 2.1. Design

This study employed a cross-sectional case–control design to investigate the association between spinal alignment and lower-limb performance during sit-to-stand (STS) activities in patients with PD compared to a control group of healthy individuals. Ethical approval for this study was obtained from the Ethical Committee of ATS Brescia (Approval No: NP3542). Prior to participation, each participant was briefed on the study’s objectives, procedures, potential risks, and benefits, and written informed consent was obtained in accordance with the Declaration of Helsinki.

### 2.2. Participants

A total of 43 consecutive patients with PD (25 males and 18 females; age 73.7 ± 7.1 years) diagnosed with idiopathic PD by a neurologist according to the UK Brain Bank criteria participated in this study [16]. These patients were enrolled during routine medical examinations in a rehabilitation department and ranged in age from 52 to 83 years (mean: 71.5 ± 7.9 years). None of the participants exhibited dyskinesias. Additionally, 42 healthy controls (22 males, 20 females; mean age 71.5 ± 7.9 years), who had no neurological diseases, were recruited through clinical examinations at Casa di Cura Domus Salutis between January 2018 and September 2019.

Inclusion criteria for both groups were ages between 60–90 years, ability to maintain a sitting position for at least 20 min, and no severe joint rigidity that could impede STS movements. Patients with PD had to have a stable pharmacological regimen for at least one month before the study. Exclusion criteria were broad to ensure clear differentiation between groups, including other neurological diagnoses, significant orthostatic hypotension, low cognitive function (Mini-Mental State Examination score below 23/30), a body mass index over 34, significant visual or neurosensory impairments, or any major health events such as hospitalization for venous thrombosis or cardiac issues within the last three months.

### 2.3. Clinical Measurements

Following the acquisition of informed consent, physical activity levels were assessed using the International Physical Activity Questionnaire (IPAQ), with energy expenditure quantified in metabolic equivalents (METs). Disease severity in patients with PD was determined using the Hoehn and Yahr scale (0–5) [21,22].

Within a multidimensional evaluation model, the Hoehn and Yahr scale (range 0–5) has been used to define the severity of disease in the “PD” group [23,24]. Afterwards, subjects underwent to a clinical examination, including body weight and standing height assessment as well as body mass index calculation [25]. This procedure was followed by a sagittal balance evaluation via plumbline distances determination at levels of C7 (PL-C7), kyphosis apex (PL-AK), L3 (PL-L3), and S1 (PL-S1). The angle of trunk rotation (ATR, expressed in degrees) was also measured with a Bunnell scoliometer™ (Orthopedic Systems, Inc., Hayward, CA, USA) in a standing, bent-over position (arms dangling, palms pressed together) [26]. The sagittal profile of the spine has also been measured by inclination assessment with a gravity-dependent inclinometer (Isomed Inc. 975 SE Sandy Blvd, Portland, OR, USA) as previously described in the literature [27].

Participants performed the Muscle Quality Index (MQI) test, which involves standing up from a seated position 10 times without the use of hands. The time taken to complete these movements was recorded, and the MQI was calculated using the formula [12]: MQI = [(lower-limb length × 0.4) × body mass × gravity × 10]/time. This test provided data on time utilized, absolute power, and relative power (Watts and Watts/Kg body weight), which were used in subsequent statistical analyses.

### 2.4. Statistics

Descriptive analysis was performed through absolute and relative frequencies for categorical variables and by using mean and standard deviation for continuous data. All clinical parameters used in this study were subjected to data distribution analysis using the Kolmogorov–Smirnov test, and normally distributed parameters are expressed as mean ± standard deviation (SD), while parameters with skewed distribution are expressed as median and interquartile range (IQR). The Student’s *t*-test and Mann–Whitney U test were used to compare the clinical, power, and spinopelvic alignment parameters between the sexes. We used the chi-square test to analyze the differences in patients with PD and controls in terms of obesity (BMI > 30), falling (at least one fall during the previous two months), the presence of at least moderate pain (VAS > 4/10), and thoracic kyphosis > 45° Cobb. Statistical significance was set at a *p*-value of <0.05. All statistical analyses were performed using IBM SPSS Statistics software (version 28.0, IBM Corp, Armonk, NY, USA).

## 3. Results

### Demographic Characteristics

This study included a total of 160 patients with PD and 170 controls, categorized by gender. Male and female patients with PD did not show significant differences in terms of age, pain, or disease duration. However, male participants, both in the PD and control groups, exhibited a significantly higher BMI compared to their female counterparts (*p* < 0.05), as outlined in Table 1.

Disease severity, as classified by the Hoehn and Yahr scale, demonstrated comparable distributions across gender within the PD cohort. Specifically, 54% of male and 48% of female patients were categorized within Hoehn and Yahr stages 0–1.5. Furthermore, 36% of males and 40% of females fell within stages 2–3. These findings indicate no statistically significant gender differences in the severity distribution of Parkinson’s disease as per the Hoehn and Yahr classification (Table 2).

Trunk rotation, measured by ATR, showed a statistically significant difference between females and males in both study groups (*p* < 0.05). The mean distances of PL-C7 on the sagittal plane was found to be significantly higher in male patient s with PD compared to their female counterparts and both sexes in controls. Also, female patients with PD presented a higher PL-C7 value than female controls (*p* < 0.05). The mean angle of upper thoracic anteflexion, as measured at C7-T2 by the digital inclinometer, showed the same pattern (*p* < 0.05). The relative power of the lower limbs (Watt/Kg), as measured by the MQI test, was lower for females in the PD group and also in control group (Table 3).

According to the study of Hasegawa et al., the two groups of patients with PD were organized into three different classes, and the threshold level of 1.7 Watt/kg was overcome by 39% of male patients with PD and by 46% of male controls, while only 13% of female patients with PD and 18% of female controls overcame this cut-off value. A total of 8% of this last group was classified below the critical level of 0.9 Watt/kg compared to only 13% of the first one. A total of 54% of male patients with PD were in the 1.0–1.6 Watt/kg range compared to 73% of their female counterparts.

## 4. Discussion

This investigation extends our prior research and, to our knowledge, represents the inaugural application of the MQI to evaluate lower-limb performance in patients with PD, specifically analyzing its association with trunk deformities. The results demonstrate a notable decrement in hip and knee extensor strength and power within the PD cohort. Historically, the MQI has been applied to assess sarcopenia and lower-limb power deficiencies in elderly populations. This study distinctively employs the MQI within a clinical context to scrutinize lower-limb power dynamics in patients with PD, thereby facilitating an examination of the consequential effects of spinal alignment on limb functionality. In alignment with the extant body of literature, our findings substantiate a significant diminution in lower-limb power among patients with PD compared to healthy controls, thereby underscoring the clinical implications of our results in the context of diagnosing and managing mobility impairments associated with Parkinson’s disease [28,29,30].

Previous studies have highlighted various postural abnormalities contributing to altered dynamic lower-limb performance in patients with PD, such as difficulties modulating responses to postural demands or delayed initiation of voluntary postural responses [31]. However, neither the MQI nor spinal sagittal trunk profiles have been previously measured in this population. Our findings reveal a positive correlation between postural abnormalities in spinal alignment, particularly at the L3 level, and sit-to-stand performance in patients with PD (*p* < 0.05). No correlations were found with other spinal parameters measured, indicating the specificity of the relationship between the L3 plumbline and MQI. This correlation aligns with previous studies suggesting a link between L3 plumbline values and spinopelvic angles in patients with PD [16]. Spinopelvic angles, in turn, correlate with lumbar lordosis and pelvic tilt angles, as observed in standard X-ray exams [17].

These correlations highlight how the reduction of lumbar lordosis in patients with PD is associated with impaired lower-limb functionality, consistent with findings by Nakamura et al., who demonstrated the correlation between lumbar lordosis values and Timed and Go-test performance [32]. The lack of correlation between lower-extremity strength and spinal alignment parameters in the control group unaffected by neurodegenerative disease suggests that patients with PD experience a specific maladaptive process involving spinal alignment and lower limbs through the lumbopelvic unit.

As anticipated from prior research, there exists a significant negative correlation between age and MQI in patients with PD, indicating a decline in lower-limb function with advancing age. However, the significant correlation observed solely in the PD group suggests that the aging process predominantly affects patients with Parkinson’s disease compared to the general population.

The absence of a standardized method by which to evaluate lower-limb performance in the PD population limits comparability with other studies. Nonetheless, the clinically oriented spinopelvic parameters measured in this patient cohort closely resemble those reported in previous research [33]. Consequently, the data on spinal alignment can be deemed representative of the average PD population observed, and we propose that the assessment protocol outlined in this study could be implemented cost-effectively in clinical settings.

Both the absolute (Watt) and relative power production (Watt/Kg) measured by the Muscle Quality Index test exhibit no correlation with the weekly METs produced in either group. This underscores the notion that non-specific physical activity does not significantly impact preventing the deterioration of muscle quality in both patients with PD and controls.

Muscle atrophy, marked by a notable decline in muscle mass and strength, along with an increase in intramuscular fat and other non-contractile components, may significantly influence both studied groups. Research has linked changes in muscle quality in the lower extremities to reduced strength in multiple studies, yet the impact of muscle quantity on lower-limb strength and spinal alignment in patients with Parkinson’s disease (PD) is less understood [34].

Developing a strength and power training regimen, which focuses on enhancing the rate of energy output over time, is essential to counteract the degenerative alterations in muscle tissue in the trunk and lower limbs [35].

Healthcare providers need to be vigilant regarding these dynamics, as the findings suggest that a notable portion of the PD cohort—about 14%—faces a potential loss of independence in everyday tasks. Additionally, 44% are at risk of exceeding the critical power-to-weight ratio of 0.9 watts/kg [36], highlighting the critical need for specialized interventions to maintain muscle functionality and prevent decline in patients with PD.

The age- and sex-corrected multivariate logic regression model shows that MQI and C7 sagittal arrow are significantly related to belonging to the PD group. In particular, an increase of 1 MQI unit is accompanied by 2% higher odds of being part of the PD group, while the increase of 1 mm C7 plumbline is accompanied by a 4% increase in the odds of being part of this group. No other variable or interaction results was significantly associated with being part of the PD group.

According to our results, in the clinical evaluation, sagittal balance is mandatory to consider data from C7, L3, S1, and kyphosis apex plumblines because the spinal alignment has to be described from a global perspective. Not only linear parameters but also angular values should be considered because in PD subjects, the upper thoracic inclination of kyphosis (T1–T2 angle) and sagittal plumblines of C7 and the kyphosis apex were significantly higher than in the control group (*p* < 0.05).

### 4.1. Implications for Clinical Practice

Our findings underscore the critical role of lumbar lordosis in sustaining lower-limb muscle biomechanics in patients with PD. Effective preservation of this spinal curvature is paramount as it significantly enhances functional capacities essential for everyday tasks such as the sit-to-stand movement. We recommend routine clinical assessments to monitor spinal alignment and adjust treatment plans proactively.

The observed gender-specific variances in spinal alignment and muscle power advocate for bespoke rehabilitation strategies. Tailoring interventions to address the unique needs of male and female patients with PD can optimize recovery outcomes and enhance the overall efficacy of rehabilitation programs.

Given the positive correlation between targeted spinal exercises and limb muscle power, integrating structured postural exercises into rehabilitation regimens is advised. These exercises should focus on enhancing spinal stability and are likely to reduce fall risks while improving mobility [37].

The MQI test, due to its efficacy and cost-effectiveness, is recommended for regular implementation in clinical settings. This tool facilitates the accurate tracking of muscle strength and power, allowing for timely adjustments in therapy to better support patient needs.

Managing PD effectively requires a comprehensive strategy involving multiple healthcare professionals. A collaborative approach that includes physical therapists, neurologists, and other relevant specialists is essential to address the diverse and complex manifestations of PD comprehensively.

We advocate for the development and implementation of specialized training programs aimed at strengthening muscle power and endurance in patients with PD. These programs should specifically target the mitigation of muscular degeneration, aiming to preserve or enhance muscle functionality and thereby improve quality of life.

### 4.2. Limits of the Study

This study has several limitations that should be considered when interpreting the findings. First, the patients with PD and the control groups were not perfectly matched by age. This discrepancy may impact the comparisons regarding the effects of PD on spinal alignment and muscle power.

Second, we employed the IPAQ to measure weekly physical activity levels. The IPAQ is not universally recognized as a reliable method for quantifying physical and recreational activities. Although all responses were collected by the same operator to minimize overestimation errors from self-administration, the potential for inaccuracies in reporting physical activity persists, presenting a challenge in accurately measuring this variable.

Additionally, the recruitment of participants through clinical examinations in a specific setting may introduce selection bias. This bias could limit the generalizability of our results to other populations or clinical environments.

Finally, the sample size was not explicitly critiqued within the provided text, but it is a crucial factor for statistical power and the validity of the conclusions. The adequacy of the sample size should be evaluated to ensure that the results are representative and reliable for the population studied.

These limitations underscore the need for a cautious interpretation of the results and suggest areas for improvement in future research to better understand the impact of PD on spinal alignment and muscle functionality.

## 5. Conclusions

This investigation reaffirms that the maintenance of lumbar lordosis is integral to preserving the biomechanical efficacy of lower-limb muscles in patients with PD. Beyond the benefits derived from general physical activities, the retention of specific physiological contractile properties in these muscles is likely better addressed through targeted multidisciplinary rehabilitation interventions. These interventions should incorporate spinal postural exercises and specially designed exercise programs aimed at enhancing lower-limb power production.

This study highlights the critical need for personalized rehabilitation strategies that account for gender-specific differences in spinal alignment and muscle power. We recommend the routine implementation of the MQI test in clinical settings due to its proven efficacy and cost-effectiveness. This practice allows for the precise monitoring of muscle strength and power, facilitating timely therapeutic adjustments that are vital for improving patient outcomes.

Effective management of PD necessitates a holistic approach involving an array of healthcare professionals. An integrated treatment model that includes physical therapists, neurologists, and other specialists is paramount to address the multifaceted nature of PD comprehensively. In summary, ensuring the preservation of lumbar lordosis not only supports the structural integrity necessary for optimal lower-limb function but also enhances the overall quality of life for patients with PD. This research reinforces the necessity of a gender-aware approach in the clinical assessment of PD and underscores the significant advantages of utilizing the MQI for ongoing clinical evaluations.

Our findings strongly advocate for the development of specialized training programs that prevent muscle degeneration and boost muscle functionality in patients with PD, specifically tailored to accommodate observed gender differences in spinal alignment and muscle strength. Such strategic interventions are essential for mitigating the progressive impairments associated with PD, ultimately fostering sustained mobility and independence in affected individuals.

## Figures and Tables

**Table 1:** Descriptive statistics of male and female patient with Parkinson’s disease and controls.

	Patients with PD				Controls			
	Male (*n* = 100)		Female (*n* = 60)		Male (*n* = 91)		Female (*n* = 79)	
	Mean	±SD	Mean	±SD	Mean	±SD	Mean	±SD
Age (years)	71.4	7.4	71.2	8.0	66.2 *	7.8	66.3	14.1
Height (cm)	170.4 ^	6.8	157.7	6.2	173.6 *^	7.2	160.0	8.1
Weight (kg)	81.8 ^	11.4	62.7	10.4	83.1 ^	14.9	63.9	12.5
BMI	28.2 ^	3.8	25.2	4.0	27.5 ^	4.3	25.1	5.0
DD (years)	7.1	5.0	6.8	5.6	-	-		
Pisa Synd	N	%	N	%	N	%	N	%
	27 ^	27	30	50	-	-	-	-

* Statistically significant at α = 0.05 PD vs. controls, sex matched. ^ Statistically significant at α = 0.05 male vs. female counterparts. BMI: body mass index; DD: disease duration; Pisa Synd: Pisa syndrome.

**Table 2:** Hoehn and Yahr distribution in male and female patients with Parkinson’s disease.

	Male Patients (*n* = 100)		Female Patients (*n* = 60)		
H and Y Class	*n*	%	*n*	%	*p*-Value
0–1/5	54	54%	29	48%	>0.05
2–3	36	36%	24	40%	>0.05
4	10	10%	7	12%	>0.05

**Table 3:** Parameters of spinal alignment in male and female patients with Parkinson’s disease.

	Patients with PD				Controls				*p*-Value
	Male (*n* = 100)		Female (*n* = 60)		Male (*n* = 91)		Female (*n* = 79)		
	Mean	±SD	Mean	±SD	Mean	±SD	Mean	±SD	
ATR (°)	3.3	4.2	4.5 ^	4.4	2.2	3.0	3.7 ^	5	<0.05
PL-C7 (mm)	122.0 *^	49.4	98.5 *	34.0	77.9	28.5	79.3	30.0	<0.05
PL-AK (mm)	28.6 *	39.0	21.6 *	22.6	7.5	13.5	11.8 ^	16.3	<0.05
PL-L3 (mm)	42.8	15.1	43.5	17.7	46.3	13.8	49.5 ^	16.5	0.15
PL-S (mm)	6.2	14.6	4.1	11.0	12.1	22.2	9.7	16.3	0.3
Incl. C7-T1 (°)	49.0 *^	10.3	53.4 *	4.7	57.9	10.1	59.5	9.5	<0.05
STS time (sec)	26.9	9.7	31.5 *	11.2	26.2	11.0	25.3	7.3	<0.05
Abs. power (W)	130.7 ^*	42.3	79.5 *	27.8	137.3^	49.1	94.0	41.6	<0.05
Rel. power (W/kg)	1.6 ^	0.5	1.3	0.4	1.7^	0.6	1.5	0.5	<0.05

* Statistically significant at α = 0.05 PD vs. controls, sex matched. ^ Statistically significant at α = 0.05 male vs. female counterparts. ATR: angle of trunk rotation; PL-C7: distance from plumbline to C7; PL-AK: distance from plumbline to kyphosis apex; PL-L3: distance form plumbline to lumbar concavity; PL-S: distance from the plumbline to the sacrum; Incl. C7-T1: inclinometer value at C7-T1; STS time: time necessary to complete the 10-times sit-to-stand test; Abs. power: absolute value of peak power determined via MQI test; Rel. power: relative value of peak power determined via MQI test.

## Data Availability

Data are contained within the article.
